# Efficient processing of abasic sites by bacterial nonhomologous end-joining Ku proteins

**DOI:** 10.1093/nar/gku1029

**Published:** 2014-10-29

**Authors:** Ana de Ory, Olga Zafra, Miguel de Vega

**Affiliations:** Instituto de Biología Molecular ‘Eladio Viñuela’ (CSIC), Centro de Biología Molecular ‘Severo Ochoa’ (CSIC-UAM). Nicolás Cabrera 1, Cantoblanco, 28049 Madrid, Spain

## Abstract

Intracellular reactive oxygen species as well as the exposure to harsh environmental conditions can cause, in the single chromosome of *Bacillus subtilis* spores, the formation of apurinic/apyrimidinic (AP) sites and strand breaks whose repair during outgrowth is crucial to guarantee cell viability. Whereas double-stranded breaks are mended by the nonhomologous end joining (NHEJ) system composed of an ATP-dependent DNA Ligase D (LigD) and the DNA-end-binding protein Ku, repair of AP sites would rely on an AP endonuclease or an AP-lyase, a polymerase and a ligase. Here we show that *B. subtilis* Ku (*Bsu*Ku), along with its pivotal role in allowing joining of two broken ends by *B. subtilis* LigD (*Bsu*LigD), is endowed with an AP/deoxyribose 5′-phosphate (5′-dRP)-lyase activity that can act on ssDNA, nicked molecules and DNA molecules without ends, suggesting a potential role in BER during spore outgrowth. Coordination with *Bsu*LigD makes possible the efficient joining of DNA ends with near terminal abasic sites. The role of this new enzymatic activity of Ku and its potential importance in the NHEJ pathway is discussed. The presence of an AP-lyase activity also in the homolog protein from the distantly related bacterium *Pseudomonas aeruginosa* allows us to expand our results to other bacterial Ku proteins.

## INTRODUCTION

DNA double-strand breaks (DSBs), the most dangerous DNA lesions as they provoke cell death or DNA rearrangements, can arise either during DNA replication or after exposition to damaging agents (1,2) their repair being crucial for the maintenance of genome integrity. Two main pathways mend these lesions: homologous recombination (HR) and nonhomologous end joining (NHEJ). In HR the information from an intact copy of the broken DNA duplex is used as template for DNA synthesis through the lesion, ensuring a faithful repair (3). Hence, HR is the main repair pathway in bacteria under conditions of rapid proliferation (4,5), but it is restricted to the S and G2 phases of the higher eukaryotic cell cycle. Contrarily, as in NHEJ the DNA ends are directly joined, the presence of a sister chromatid to act as template for DNA synthesis is unnecessary (6). For that reason, this pathway is active through the eukaryotic cell cycle (7–10), as well as in many bacterial species that spend much of their life cycle in stationary phase during which only a single copy of their chromosome is present (5,11). In NHEJ, the ends are usually processed by nucleolytic and/or polymerization activities before the final sealing, a fact that often leads to a mutagenic repair [reviewed in (12)].

In higher eukaryotes NHEJ is started by the binding to the DNA ends of the Ku70/80 heterodimer that together with the DNA-dependent protein kinase catalytic subunit (13,14) bring about the synapsis of the broken ends. Further recruitment of nucleases (Artemis, Fen1) and/or polymerases (λ, μ and yeast Pol IV) (15–19) causes the processing of the ends that are finally ligated by the complex Ligase IV/XRCC4/XLF [reviewed in (7)].

In prokaryotes, the NHEJ complex is composed by a Ku homodimer and a dedicated ligase (LigD), a multidomain protein that in most cases contains phosphoesterase, polymerase and ligase activities that account for the end processing, gap filling and sealing steps [reviewed in (4,5)]. Recently, the presence of a bacterial related NHEJ complex has been reported also in archaea and constituted by Ku, and stand-alone ligase, polymerase and phosphoesterase proteins (20).

In both bacterial and eukaryotic NHEJ, Ku plays a pivotal role by threading the broken ends through its central DNA binding ring, allowing their alignment. Previous identification of prokaryotic Ku homologs (21) and later resolution of the human Ku heterodimer structure (22) allowed to dissect the protein into three distinct domains. Thus, the central ring-shaped core, conserved in eukaryotes and prokaryotes, contains the regions responsible for dimerization and for encircling duplex DNA. The von Willebrand A domain, specifically fused to the N-terminus of the core in the eukaryotic Ku, mediates protein–protein interactions (21–23). Finally, the eukaryotic Ku70 subunit has a C-terminal extension similar to SAP domains involved in DNA binding, the only bacterial homolog with a SAP-like domain being the Ku protein from *Streptomyces coelicolor* (21,22).

Although the role of eukaryotic Ku in NHEJ had been previously assumed only to recognize broken DNA ends and to recruit the above-mentioned factors, an unexpected primary role in end processing has been recently demonstrated (24). Thus, human Ku possesses an intrinsic AP lyase activity essential for the efficient removal of AP sites near DSBs to allow final joining of those damaged ends (24,25). Such an activity was mapped at the N-terminal von Willebrand A domain of the Ku70 subunit, therefore the presence of an AP-lyase in a bacterial Ku could not be anticipated. Thus, notwithstanding that bacterial Ku has been also studied in members of the genera *Bacillus*, *Mycobacterium* and *Pseudomonas*, no additional roles other than DNA binding and recruitment of LigD have been identified so far (26–33).

In this paper we show that *Bacillus subtilis* Ku (*Bsu*Ku), is endowed with an intrinsic AP/5′-dRP lyase activity. The *Bsu*Ku–LigD complex coordinates efficient processing of DNA ends with near terminal abasic sites to allow the repair of DSBs through the NHEJ pathway.

## MATERIAL AND METHODS

### Proteins and reagents

Unlabeled nucleotides were purchased from GE Healthcare. Labeled nucleotides were obtained from Perkin Elmer Life Sciences. Substrates were radiolabeled at the 3′ end with [α^32^P]-Cordycepin (3′-dATP) and terminal deoxynucleotidyl transferase (TdT) or at the 5′ end with [γ ^32^P]ATP and T4 polynucleotide kinase (T4PNK). TdT, T4PNK, *h*APE1, *Escherichia coli* Uracil DNA Glycosylase (UDG), *E. coli* EndoIII, nickase Nt.BStNBI, T4 DNA ligase and restriction enzymes AatII, HindIII and SmaI were obtained from New England Biolabs. Thrombin was from Novagen. Streptavidin was from Sigma-Aldrich. *Bsu*Ku and *Bsu*LigD were purified as described (26). The purified *Bsu*Ku was further loaded onto a 4 ml glycerol gradient (15–30%) containing 30 mM Hepes, pH 7.4, 20 mM ammonium sulfate, 180 mM NaCl, 1 mM EDTA, and 7 mM β-mercaptoethanol and centrifuged at 4°C for 24 h at 348 134 x *g* in a Beckman TST 60.4 Swinging rotor. After centrifugation, 24 fractions were collected from the bottom of the tube for further analysis. Five microliters of each fraction were analysed in the AP-lyase activity (see below).

Cloning, overexpression and purification of *Pseudomonas aeruginosa* Ku (*Pae*Ku). A DNA encoding *Pae*Ku (21) was PCR-amplified from *P. aeruginosa* strain PAO-1 genomic DNA using forward (5′-CGCGCGGGCATATGGCGCGTGCGATCTGGAAAGG) and reverse (5′-GGCCGGATCCTCAGGCCTTGCGCCGCGAACG) primers. The forward and reverse primer contained an NdeI and BamHI site, respectively. Therefore, the amplified DNA was digested with NdeI and BamHI before cloning in the NdeI-BamHI digested pET-28a(+) bacterial expression vector (Novagen), which carries an N-terminal His-Tag configuration to express the recombinant protein as fusions with an N-terminal hexahistidyl for purification on Ni^2+^-affinity resins. The insert was sequenced completely to rule out the acquisition of mutations during amplification and cloning. *Escherichia coli* BL21(DE3) cells were transformed with the recombinant expression plasmid pET-28PaeKu, and such construction was further confirmed by DNA sequencing. Expression of the His-tagged *Pae*Ku protein was carried out in the *E. coli* strain BL21(DE3), which contains the T7 RNA polymerase gene under the control of the isopropyl β-D-thiogalactopyranoside (IPTG)-inducible lacUV5 promoter (34,35). Cells, previously transformed with plasmid pET-28PaeKu were grown overnight in LB medium at 37°C in the presence of kanamycin. Cells were diluted into the same media and incubated at 34°C until the DO_600_ reached 0.6. Then, IPTG (Sigma) was added to a final concentration of 0.5 mM. After additional 1 h at 30°C, 50 μg/ml of rifampicin was added and incubation was continued for 90 min at 30°C. Cells were thawed and ground with alumina at 4°C. The slurry was resuspended in Buffer B (50 mM Tris-HCl, pH7.5, 0.7 M NaCl, 7 mM β-mercaptoethanol, 1 mM EDTA, 5% glycerol) and centrifuged for 5 min at 650 x *g*, at 4°C to remove alumina and intact cells. The recombinant *Pae*Ku protein was soluble under these conditions, since it remained in the supernatant after a new centrifugation for 20 min at 23 430 x *g*, to separate insoluble proteins from the soluble extract. The DNA present was removed by stirring for 10 min the soluble extract containing 0.3% polyethyleneimine (PEI) followed by centrifugation for 10 min at 23 430 x *g*. The resulting supernatant was precipitated with ammonium sulfate to 65% saturation to obtain a PEI-free protein pellet. After centrifugation for 25 min at 23 430 x *g*, the pellet was resuspended in Buffer B 0.25 M NaCl. This fraction was loaded onto a phosphocellulose column equilibrated with Buffer B (0.25 M NaCl) and the *Pae*Ku protein was eluted with Buffer A (0.5 M NaCl). The eluate was loaded onto a Ni-NTA column (Qiagen) pre-equilibrated with Buffer A (0.5 M NaCl). The bound protein was eluted by 200 mM imidazole in Buffer A (0.3 M NaCl). Finally, *Pae*Ku was dialyzed against a buffer containing 0.3 M NaCl and 50% glycerol and stored at -20°C. Final purity of the protein was estimated to be >90 % by SDS-PAGE followed by Coomassie blue staining.

### DNA substrates

Oligonucleotides H (5′-GTACCCGGGGATCCGTACHGCGCATCAGCTGCAG) where H stands for tetrahydrofuran (THF); pU (5′-CTGCAGCTGATGCGCUGTACGGATCCCCGGGTAC); pG (5′-GTACCCGGGGATCCGTACGGCGCATCAGCTGCAG); U4 (5′- TCAUACTGATCACAGTGAGTAC); sp1cddC (5′-GTACTCACTGTGATddC) where ddC stands for 2′,3′-dideoxyCMP; U4c (5′- AGTGATCACAGTGAGTAC); sp1c (5′-GTACTCACTGTGATC); sp1c-1 (5′- GTACTCACTGTGAT); sp1CGG (5′- GATCACAGTGAGTACCGG); sp1GCC (5′-GATCACAGTGAGTACGCC), sp1TCC (5′-GATCACAGTGAGTACTCC); sp1ACC (5′-GATCACAGTGAGTACACC); sp1c(p) (5′-pGTACTCACTGTGATC); U2 (5′-TUAGACTGATCACAGTGAGTAC); U19 (5′- GATCACAGTGAGTACTCAUACT) and U21 (5′-GATCACAGTGAGTACTCAGAUT) were obtained from Invitrogen. Oligonucleotides sp1 (5′-GATCACAGTGAGTAC); Dws 5′-P (5′-P-AACGACGGCCAGT), sp1c+13 (5′-ACTGGCCGTCGTTGTACTCACTGTGATC), SU (5′-GCTTCCGGTGATCCGTTCCGTGAGGTCTCGACATGCGCUGTACGATTGCGCTAGTACACTGGCCGACGTTTGTACACTGT) and SU-compl (5′-biotin-ACAGTGTACAAACGTCGGCCAGTGTACTAGCGCAATCGTACAGCGCATGTCGAGACCTCACGGAACGGATCACCGGAAGC-biotinTEG) were obtained from Sigma-Aldrich. pU was hybridized with pG to obtain a blunt substrate. SU was hybridized to SU-compl to obtain a dsDNA 80 bp long. U4 was hybridized with sp1cddC to obtain a 5′-protruding structure. U4c was hybridized with sp1c or sp1c-1 to obtain two different 5′-protruding substrates. sp1CGG was hybridized to sp1c to obtain a dsDNA molecule with a CGG protruding 3′ end. sp1GCC, sp1TCC and sp1ACC were hybridized to the complementary sp1c(p) to obtain dsDNA molecules with -GCC, -TCC and -ACC protruding 3′ ends, respectively. U2, U4, U19 and U21 were hybridized to sp1c to obtain 5′ (U2/U4) or 3′ (U19/U21) protruding ends. Nicked substrate was made by hybridizing oligonucleotides sp1 and Dws 5′-P to sp1c+13. All the hybridizations were performed in the presence of 0.2 M NaCl. A 216 bp DNA fragment corresponding to *B. subtilis* gene *yshC* was PCR-amplified using as template genomic DNA, 5-labeled and used as substrate in DNA gel retardation assays (see below). All the experiments presented were repeated at least three times.

### AP/5′-dRP lyase activity assay on uracil containing substrates

Analysis of the AP-lyase activity on ssDNA was evaluated with the 34-mer oligonucleotide pU, labeled at the indicated 3′ or 5′ end. Activity on dsDNA was gauged on the 3′ labeled-pU/pG hybrid. The ability of *Bsu*Ku to act on AP sites placed at 5′ protruding ends was studied using as substrates the 3′ labeled-U2/sp1c and 3′ labeled-U4/sp1c hybrids, whereas the activity on 3′ protruding ends was evaluated with the 5′ labeled-U19/sp1c and 5′ labeled-U21/sp1c hybrids, as indicated. Those uracil-containing molecules were treated with *E. coli* UDG (0.2 U) for 15 min at 37°C in the presence of 30 mM Hepes, pH 7.5, 4% glycerol. After incubation, the mixture was supplemented with 0.2 U *h*APE1, 2 U of Endo III or the indicated concentration of either *Bsu*Ku or *Pae*Ku. Samples were incubated at 30°C for the indicated times and reactions were stopped by addition of freshly prepared NaBH_4_ to a final concentration of 100 mM and incubation on ice for an additional 20 min. DNA was ethanol-precipitated in the presence of 0.2 μg/ml tRNA. Reactions were analysed by 8 M urea-20% PAGE and autoradiography. Analysis of the activity on THF containing DNA was performed as described above using oligonucleotide H that harbors a THF at position 19.

To analyse the 5′-dRP lyase activity, 3′-radiolabeled substrate pU was hybridized to oligonucleotide pG to obtain a 34-mer dsDNA substrate. Reactions (12.5 μl) contained 30 mM Hepes, pH 7.5, 4% glycerol (v/v), 0.2 U *E. coli* UDG and 0.2 U *h*APE1. Reactions were initiated by adding 228 nM of either purified *Bsu*Ku or Pol λ as indicated. Samples were incubated at 30°C for 30 min. The reactions were stopped by addition of freshly prepared NaBH_4_ to a final concentration of 100 mM and further incubated for additional 20 min on ice. DNA was ethanol-precipitated in the presence of 0.2 μg/ml tRNA and analysed by 8 M urea-20% PAGE and autoradiography.

Analysis of kinetics was carried out as described in (24), by incubating saturating concentrations of *Bsu*Ku with the above-described substrates at 30°C and the fraction of product (y) generated at various times (x) was determined by electrophoresis. Values for kobs were determined by a nonlinear regression fitting of the data to the equation y = ymax(1-e^−kobs*x^) (Prism, Graphpad software).

### NaBH_4_ trapping assay

The Schiff base intermediate formed between an AP site and purified *Bsu*Ku was trapped by the addition of 100 mM NaBH_4_ or 100 mM NaCl immediately after adding 228 nM of *Bsu*Ku to 3.1 nM of deglycosylated 3′-labeled oligonucleotide pU (see above). When indicated, and to remove the N-terminal His-tag, the purified *Bsu*Ku was preincubated with thrombin (0.05 U) in its reaction buffer for 1 h at 20°C in a total volume of 15 μl. The reactions were performed in a total volume of 25 μl in the presence of 30 mM Hepes, pH 7.5 and 4% glycerol. After incubation for 30 min at 37°C, samples were analysed by 12% SDS-PAGE followed by Coomassie blue staining and autoradiography of the dried gel.

### Agarose gel retardation

Two micrograms of supercoiled pUC19 plasmid (Fermentas) were incubated with 10 U of the nickase Nt.BStNBI to obtain nicked pUC19. The DNA-binding assay was performed by incubating the indicated amounts of *Bsu*Ku with 100 ng of either supercoiled or nicked pUC19 in buffer containing 50 mM Hepes, pH 7.5, 4% glycerol (v/v), in a final volume of 10 μl for 60 min at room temperature. When indicated the nucleoprotein complex was dissociated by proteinase K digestion for 1 h at 50°C in the presence of 0.2% SDS. Complexes were resolved on 0.7% TAE-agarose gels and visualized with ethidium bromide staining.

### AP-lyase activity at internal abasic sites

AP-lyase activity at internal abasic sites generated in circular DNAs. AP sites were generated essentially as described (36). Briefly, negatively supercoiled plasmids pUC19 and pcDNA3.1 (Invitrogen) were incubated in 50 μl of 25 mM sodium citrate, pH 4.8, 250 mM KCl at 70°C for 1 h. Reactions were stopped by addition of 50 μl of precooled 1 M Tris-HCl, pH 8, followed by buffer exchange into water in a Qiaquik spin column (Qiagen). The AP-lyase assay was carried out in a final volume of 10 μl containing 50 mM Hepes, pH 7.4, 4% glycerol (v/v) and 10 mM MgCl_2_, 100 ng of the nontreated (pcDNA3.1 and pUC19) or AP-containing plasmids (pcDNA3.1-AP and pUC19-AP) with either 10 U of *h*Ape 1, 10 U of EcoRI, 100 ng of BSA or the indicated amount of *Bsu*Ku. Samples were incubated for 30 min at 30°C and further incubated with 10 μg of proteinase K at 50°C for 1 h in the presence of 0.2% SDS. The reaction products were resolved on 0.7% TAE-agarose gels and visualized with ethidium bromide staining.AP-lyase activity at an internal abasic site in a linear DNA. The 3′-labeled oligonucleotide SU, which contains a uracil at position 40 was hybridized to the complementary oligonucleotide SU-comp harboring a 5′-biotin and a 3′-biotin TEG modification, and 3.2 nM of this uracil-containing molecule were treated with *E. coli* UDG (0.2 U) for 15 min at 37°C in the presence of 30 mM Hepes, pH 7.5, 4% glycerol, in the absence or presence of 200 nM streptavidin. After incubation, the mixture was supplemented with the indicated concentration of *Bsu*Ku and further incubated at 30°C for the indicated times. Reactions were stopped by addition of 10 μg of proteinase K and further incubation at 50°C for 1 h in the presence of 0.4% SDS. Reactions were analysed by 8 M urea-20% PAGE and autoradiography.

### Electrophoretic mobility shift assay (EMSA)

The incubation mixture contained, in a final volume of 20 μl, 12 mM Tris-HCl, pH 7.5, 4% glycerol (v/v), 1 mM EDTA, 20 mM ammonium sulfate, 0.1 mg/ml BSA, 1 nM of either the labeled 5 nucleotides gapped DNA structure or a 5′-labeled 216 bp DNA fragment and the indicated amounts of the specified protein. For the supershift reactions, DNA was preincubated for 10 min at 4°C with 3 nM of *Bsu*Ku followed by the addition of the indicated concentrations of *Bsu*LigD. After incubation for 10 min at 4°C, the samples were subjected to electrophoresis in precooled 4% (w/v) polyacrylamide gels (80:1, monomer:bis) containing 12 mM Tris-acetate, pH 7.5 and 1 mM EDTA, and run at 4ºC in the same buffer at 8 V/cm (37). After autoradiography, protein–DNA complexes were detected as a shift (retardation) in the migrating position of the labeled DNA.

### End-joining of linearized plasmids

The reaction mixture contained, in a final volume of 20 μl, 12 mM Tris-HCl, pH 7.5, 4% glycerol (v/v), 1 mM EDTA, 20 mM ammonium sulfate, 0.1 mg/ml BSA, 100 ng of the indicated linearized plasmid (HindIII-, AatII- or SmaI-digested pUC19 plasmid), 0.2 mM ATP, 0.64 mM MnCl_2_, 100 ng of *Bsu*LigD and/or 100 ng of *Bsu*Ku, when indicated. After incubation for 60 min at 30°C, reactions were quenched by adjusting the mixtures to 0.2% SDS, 10 mM EDTA. Samples were incubated with 10 μg of proteinase K during 60 min at 50°C and subjected to 0.7% agarose gel electrophoresis.

### End joining of partially complementary 3′-protruding DNA ends

The reaction mixture contained, in a final volume of 12.5 μl, 12 mM Tris-HCl, pH 7.5, 1 mM EDTA, 20 mM ammonium sulfate, 0.1 mg/ml BSA, 1.25 nM of the labeled sp1CGG/sp1c hybrid and 2.5 nM of each of the indicated nonlabeled DNA molecule containing a protruding 3′ end, 71 nM of both *Bsu*LigD and *Bsu*Ku, 0.64 mM MnCl_2_ and 100 nM of the indicated NTP. After incubation for 30 min at 30°C, the reactions were stopped by adding EDTA up to 10 mM. Samples were analysed by 8 M urea, 20% PAGE and autoradiography.

### End joining of partially complementary DNA ends with near terminal AP sites

The 3′-labeled substrate U4 was hybridized to sp1cddC to obtain a 7 nt 5′-protruding DNA (substrate *A* in Figure 9). This substrate was deglycosylated with *E.coli* UDG, as described above to obtain an AP site in the protruding 5′-end. When indicated, the deglycosylated DNA was incubated in the presence of *h*APE1 to produce a 5′-dRP containing substrate at the protruding 5′-end. The 0.68 nM of each of the above processed substrates were incubated in a reaction volume of 15 μl in the presence of 30 mM Hepes pH, 7.5, 4% glycerol (v/v), 114 nM of purified *Bsu*Ku and/or 114 nM of purified *Bsu*LigD, 0.64 mM MnCl_2_, 1 nM of either the nonlabeled hybrid sp1c/U4c (upstream substrate *D* in Figure 9) or the nonlabeled hybrid sp1c-1/U4c (upstream substrate *E* in Figure 9), and 10 μM of the indicated nucleotide. Samples were incubated at 30°C for 30 min. The reactions were stopped by addition of freshly prepared NaBH_4_ to a final concentration of 100 mM and incubated 20 min on ice. DNA was ethanol-precipitated in the presence of 0.2 μg/ml tRNA. Reactions were analysed by 8 M urea–20% PAGE and autoradiography.

**Figure 1.**
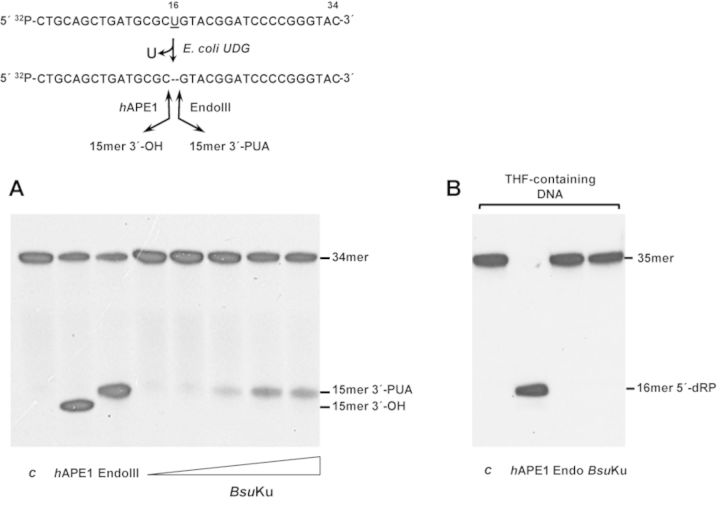
*Bsu*Ku is endowed with AP-lyase activity. (**A**) The [^32^P]5′-labeled uracil-containing oligonucleotide was treated with *Escherichia coli* UDG, leaving an intact AP site. The resulting AP-containing DNA was incubated in the presence of either *h*APE1 that cleaves 5′ to the AP site, EndoIII that incises 3′ to the AP site or increasing concentrations (9, 18, 36, 72 and 142 nM) of *Bsu*Ku for 1 h at 30°C, as described in Materials and Methods. *c*: control DNA after treatment with *E. coli* UDG. After incubation samples were analysed by 8 M urea-20% PAGE and autoradiography. Position of products is indicated. (**B**) The 3′[^32^P]3′-dAMP labeled oligonucleotide containing the lyase-resistant analog tetrahydrofuran (THF) at position 19 was incubated in the presence of either *h*APE1, EndoIII or *Bsu*Ku (228 nM) as described above. *c*: control DNA incubated in the absence of proteins. Position corresponding to product (16mer 5′-dRP) is indicated.

**Figure 2.**
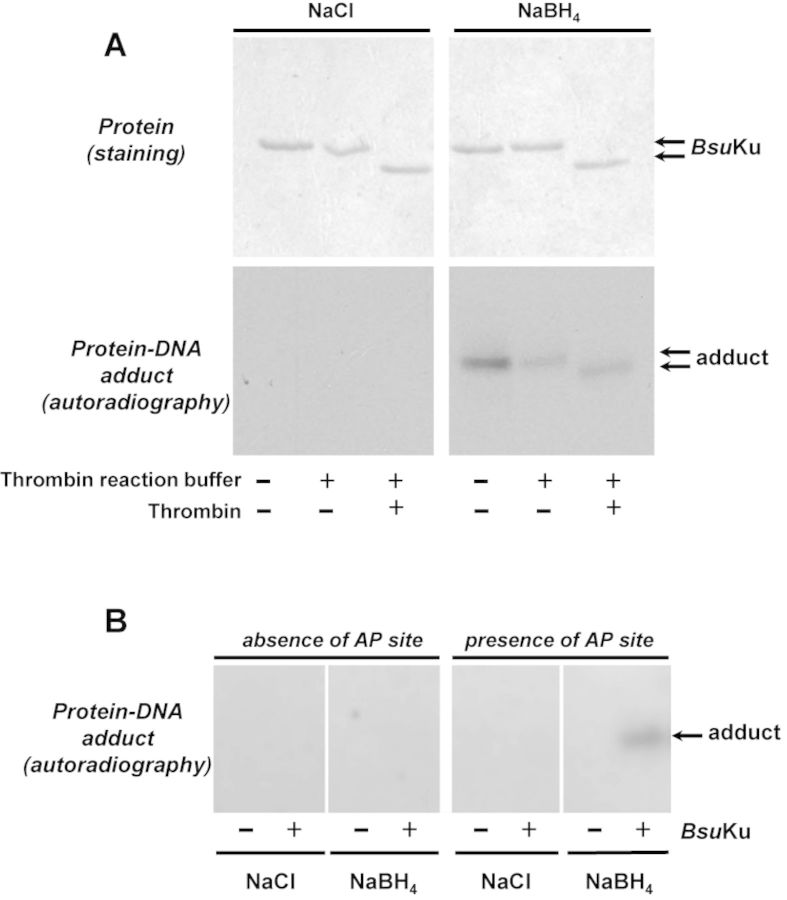
Formation of *Bsu*Ku-DNA adducts. (**A**) Dependence of *Bsu*Ku-DNA cross-link on NaBH_4_. Reactions were performed as described in Materials and Methods, incubating 228 nM *Bsu*Ku with 3.1 nM of the 3′[^32^P]3′-dAMP labeled 35mer oligonucleotide containing an AP site at position 16 (after treatment with *Escherichia coli* UDG), in the presence of either 100 mM NaBH_4_ or NaCl (as indicated). *Top panels*, Coomassie blue staining after SDS-PAGE of purified *Bsu*Ku. *Bottom panels*, autoradiography of corresponding protein–DNA adducts after the SDS-PAGE separation shown in top panels. When indicated, protein was previously incubated with 0.05 U of thrombin at 20°C for 60 min. (**B**) Adduct formation is dependent on the presence of an abasic site. Reactions were performed as described in (A) but using as substrate 3.1 nM of the 3′[^32^P]3′-dAMP labeled 35mer oligonucleotide without removing the uracil at position 16 (absence of AP site) or after treatment with *E. coli* UDG (presence of AP site), in the presence of either 100 mM NaBH_4_ or NaCl (as indicated). Autoradiography of corresponding protein–DNA adduct after the SDS-PAGE separation is shown.

## RESULTS AND DISCUSSION

### *Bsu*Ku is endowed with an AP/dRP-lyase activity

DNA repair proteins endowed with an AP lyase activity recognize AP sites and incise at their 3′ side through a nonmetal-dependent β-elimination reaction, resulting in strand breaks harboring a 3′-phospho-α,β-unsaturated aldehyde (3′-PUA) end that should be cleaned to allow subsequent repair reactions to occur. Previous studies in human Ku led us to evaluate the ability of bacterial Ku to recognize and incise an internal AP site. To this end, a 34-mer ssDNA containing a uracil at position 16 was previously treated with *E. coli* UDG to get a natural AP site (see Materials and Methods and scheme in Figure 1). As expected, incubation of this substrate with *h*APE1 and Mn^2+^ ions produced a 15-mer product with a 3′-OH end (Figure 1A) as this enzyme is a metal-dependent AP endonuclease that hydrolyzes the phosphodiester bond 5′ to the AP site [(38) and references therein]. In contrast, incision at the 3′ side by the AP-lyase activity of *E. coli* Endo III left a product that migrates slower due to the presence of the resulting 3′-PUA [(38) and references therein]. As observed in Figure 1A, incubation of the AP site containing DNA with increasing amounts of highly purified *Bsu*Ku in the absence of divalent cations rendered a nicked product with identical electrophoretical mobility to those produced by Endo III and with an observed rate (*k_obs_*) of 0.011 ± 1.5 × 10^−3^ min^−1^. These results are consistent with cleavage 3′ to the AP site in a metal-independent manner, leading us to infer the presence of an intrinsic AP-lyase activity in *Bsu*Ku, active also on AP-containing dsDNA substrates, although much less efficiently than on ssDNA molecules (*k_obs_* = 5 × 10^−3^ ± 5 × 10^−4^ min^−1^) (see Supplementary Figure S1). Furthermore, the results suggest that *Bsu*Ku exerts its AP lyase activity through a β-elimination reaction since if it had been through a β,δ-mechanism the products released should have migrated even faster than those produced by *h*APE1 due to the presence of a 3′ phosphate group (39).

The aforementioned conclusion was supported by the inhibition of *Bsu*Ku activity when the AP site was replaced with tetrahydrofuran (THF), a stable AP analog resistant to the β-elimination reaction (Figure 1B) (39,40). Additionally, it was analysed whether a covalent *Bsu*Ku–DNA complex could be trapped by chemical reduction as it occurs with other enzymes that proceed through a β-elimination mechanism (41). During AP site cleavage the ϵ-NH_2_ of the nucleophilic lysine forms a Schiff base with the C1′ atom of the deoxyribose. This intermediate can be reduced *in vitro* by sodium borohydride (NaBH_4_) treatment, making an irreversible covalent enzyme–DNA adduct (39,42–45). As shown in Figure 2A, *Bsu*Ku forms a stable adduct with the 3′ labeled AP site-containing 35mer ssDNA that was dependent on both, addition of NaBH_4_ as no radioactive bands were detected when the protein was incubated with an equimolar concentration of NaCl, and presence of an AP site in the ssDNA (Figure 2B). Removal of the fused N-terminal His-tag from *Bsu*Ku after incubation with thrombin gave rise to DNA–*Bsu*Ku adducts whose faster migration paralleled the electrophoretical pattern of the purified protein, a result that serves to unequivocally assign the observed AP-lyase activity to *Bsu*Ku (Figure 2A). Additionally, the purified protein was sedimented through a glycerol gradient, and the collected fractions were individually assayed for AP-lyase activity (see Materials and Methods). As shown in Supplementary Figure S2, the maximum activity coincided with the protein peak, identified by Coomassie Blue staining after SDS-PAGE analysis of each gradient fraction. These results allowed us to ascribe the AP-lyase activity to *Bsu*Ku, ruling out the presence of a contaminant AP lyase from the expression bacteria *E. coli*.

**Figure 3.**
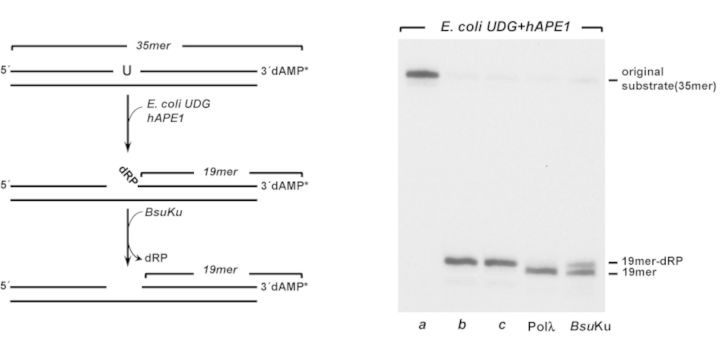
5′-dRP lyase activity of *Bsu*Ku. *In vitro* reconstitution of single-nucleotide BER. *Left*, schematic representation of a reconstituted BER reaction, indicating the different products formed. *Right*, autoradiogram illustrating *Bsu*Ku 5′-dRP lyase activity. *a*, original substrate; *b*, 5′-dRP-19mer product obtained after incubation with *Escherichia coli* UDG and *h*APE1; *c*, 5′-dRP-19mer product incubated for 30 min at 30°C. Reactions were carried out as described in Materials and Methods in the presence of 228 nM *Bsu*Ku or Pol λ, as indicated.

The presence of an AP-lyase activity in *Bsu*Ku led us to analyse the ability of the protein to excise the 5′-dRP moiety as those that results after hydrolysis of an AP site by a class II endonuclease during the first steps of the BER pathway. To this end, a 35-mer double-stranded oligonucleotide, containing a dUMP at position 16 of the 3′-labeled strand (see scheme in Figure 3 and lane *a*) was treated with *E. coli* UDG to render an AP site. Further incubation with *h*APE1 released a nicked molecule whose 3′ product contains a 5′-dRP end (see scheme and lane *b* in Figure 3) that remained stable throughout the assay (lane *c*). As a control, substrate was subjected to the well-characterized 5′-dRP lyase activity of Pol λ (46). As shown, *Bsu*Ku was able to remove the 5′-dRP group, as deduced from the reduction in the size of the 3′-labeled substrate (*k_obs_* = 0.06 ± 0.02 min^−1^).

**Figure 4.**
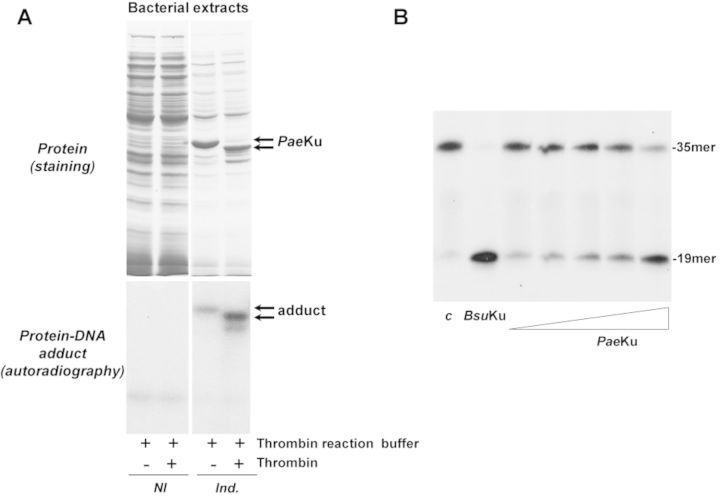
AP-lyase activity is also present in the Ku protein from the Gram- bacteria *P. aeruginosa*. (**A**) Formation of *Pae*Ku–DNA adducts. *Top panels*, expression of the recombinant *Pae*Ku. For a better visualization of the induced *Pae*Ku, 15 and 6 μg of total protein from the cellular extracts were loaded in the noninduced (NI) and in the induced (Ind.) lanes, respectively. *Bottom panels*, autoradiography of corresponding protein–DNA adducts after the SDS-PAGE separation. Reactions were performed as described in Materials and Methods, incubating either 1.7 μg of the noninduced bacterial extract or 0.7 μg of the induced extract with 3.1 nM of the 3′[^32^P]3′-dAMP labeled 35mer oligonucleotide containing an AP site at position 16 (after treatment with *Escherichia coli* UDG), in the presence of 100 mM NaBH_4_. When indicated, the bacterial extracts were previously incubated with 0.05 U of thrombin at 20°C for 60 min. (**B**) AP-lyase activity of purified *Pae*Ku. 3.1 nM of the 3′[^32^P]3′-dAMP labeled 35mer oligonucleotide containing an AP site at position 16 (after treatment with *E. coli* UDG) was incubated in the presence of either *Bsu*Ku (228 nM), or increasing concentrations (14, 28, 57, 114 and 228 nM) of *Pae*Ku for 1 h at 30°C, as described in Materials and Methods. *c*: control DNA after treatment with *E. coli* UDG. After incubation, samples were analysed by 8 M urea-20% PAGE and autoradiography. Position of products is indicated.

The unexpected presence of an AP-lyase activity in *Bsu*Ku led us to study whether this activity is specific to the *B. subtilis* protein or, by the contrary is present in other bacterial Ku. To this end, similar assays were performed with the Ku ortholog from the Gram- bacteria *Pseudomonas aeruginosa* (*Pae*Ku) (see Materials and Methods) as this organism has been used as model for the bacterial NHEJ for years (4,5). *Pae*Ku was cloned in expression plasmid pET28a and expressed in the *E.coli* BL21(DE)3 strain (see Materials and Methods). As shown in Figure 4A, expression of *Pae*Ku gives rise to a protein–DNA adduct after reduction with NaBH4, absent with the noninduced bacterial extracts. The different mobility of the adduct after digestion with thrombin demonstrates that the expressed *Pae*Ku is the protein crosslinked to the AP-containing DNA. As observed in Figure 4B, incubation of the AP site containing DNA with increasing amounts of the purified *Pae*Ku in the absence of divalent cations rendered a nicked product with the same electrophoretical mobility to that produced by *Bsu*Ku. These results allow us to extend the observation of an AP-lyase activity to other bacterial members.

### *Bsu*Ku can nick internal AP sites in a DNA without free ends

**Figure 5.**
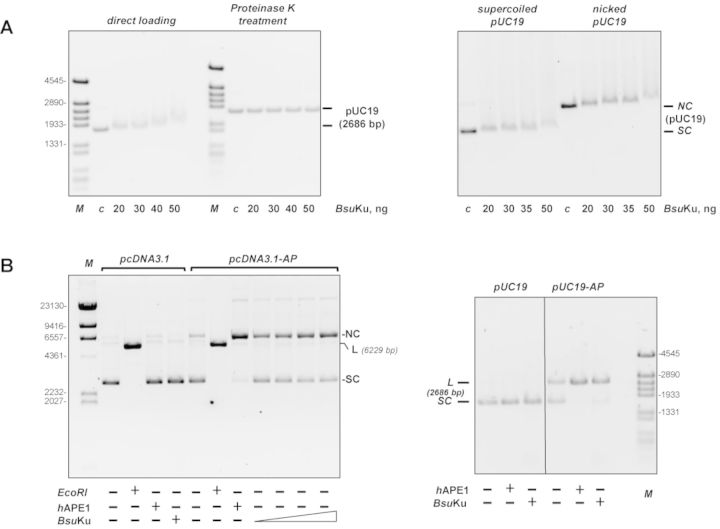
*Bsu*Ku binds circular DNA. (**A**) *Left panel*, *Bsu*Ku binding to covalently closed pUC19 plasmid. The assay was performed as described in Materials and Methods incubating the indicated amounts of *Bsu*Ku with 100 ng of supercoiled plasmid pUC19. Products were analysed on 0.7% agarose electrophoresis. One half of each reaction mixture was loaded directly onto the agarose gel (direct loading lanes), while the other half was being treated with Proteinase K. After proteolytic digestion, those samples were loaded in the same agarose gel (Proteinase K treatment lanes). The electrophoretical mobility of the pUC19 plasmid in each case is indicated. In lane c, DNA was incubated with 50 ng of BSA. *Right panel*, *Bsu*Ku binding to supercoiled and relaxed plasmid DNA. The assay was performed essentially as described above. (**B**) *Bsu*Ku AP lyase activity can act on circular substrates. The assay was performed as described in Materials and Methods, incubating either 142 nM of *Bsu*Ku with 100 ng of plasmid without AP sites (pcDNA3.1) or 18, 36, 72 and 142 nM of *Bsu*Ku with 100 ng of plasmid containing AP sites (pcDNA3.1-AP) (left panel). The absence and presence of the AP sites in pcDNA3.1 and pcDNA3.1-AP, respectively, was confirmed after digestion with *h*APE1. As a control of the linear form, plasmids were also digested with EcoRI. *NC*: nicked circles; *SC*: supercoiled; *L*: linear. The assay shown in the right panel was performed by incubating 100 ng of plasmid without (pUC19) or with (pUC19-AP) AP sites with either *h*APE1 or 142 nM of *Bsu*Ku, as indicated. Figure 5B, right panel is a composite image made from different parts of the same experiment.

Considering the absence of DNA ends in the bacterial genome, processing of abasic sites by *Bsu*Ku would require binding of the protein to internal sites of the chromosome. Hence, to ascertain the potential ability of *Bsu*Ku to associate with a DNA without free ends, we analysed the binding of the protein to a supercoiled plasmid DNA. As shown in the left panel of Figure 5A, addition of increasing amounts of *Bsu*Ku resulted in a reduction in the electrophoretical mobility of the DNA, indicating that the protein binds to internal sites of the superhelical plasmid. Such a binding is not an artifact of a conformational change of the plasmid due to a possible nicking activity as the substrate recovered its original form upon treatment with proteinase K. Similarly, *Bsu*Ku was also able to form protein–DNA complexes with a relaxed circular DNA (Figure 5, right panel). Hence, binding of *Bsu*Ku to supercoiled DNA does not depend on the presence of potential extrusion of hairpin structures. These results suggest that besides the ‘canonical’ binding mode in which the dsDNA is threaded through the central ring, *Bsu*Ku shows an alternative binding manner, most probably through positive charges located at the surface of the protein, as suggested for *Mycobacterium smegmatis* Ku (29).

The ability to bind internal sites appears to be a common feature of Ku. In this sense, eukaryotic Ku protein has been shown to bind, besides to DNA ends, to internal sites of the chromosome as to nicks, to structural transition as well as to replication origins (47,48). In addition, bacterial Ku homologs, as that from *M. smegmatis*, have been recently shown to also bind circular plasmids (29). Therefore, once established the ability of *Bsu*Ku to bind a DNA molecule lacking free ends, we analysed the capacity of the AP-lyase activity to recognize and cleavage the internal AP sites generated in a circular plasmid [(pcDNA3.1-AP); see Materials and Methods]. The absence (in pcDNA3.1) and presence (in pcDNA3.1-AP) of AP sites was checked by incubation of these plasmids with *h*APE1. Thus, as shown in the left panel of Figure 5B, nearly all pcDNA3.1-AP molecules contained at least one AP site as deduced from the conversion of the supercoiled molecules to the open form (nicked circles) after treatment with *h*APE1. Importantly, *Bsu*Ku also relaxed the supercoiled plasmid pcDNA3.1-AP, indicating that the protein recognizes and introduces nicks at the AP sites through its inherent AP-lyase activity. A similar result was obtained with a pUC19 plasmid (Figure 5B, right panel). In this case, both *h*APE1 and *Bsu*Ku linearized the supercoiled plasmid most probably due to the proximity of two AP sites in opposite strands. Similar results were obtained on a 3′-,5′-biotinylated linear dsDNA 80 bp long with streptavidin-blocked ends to prevent *Bsu*Ku from sliding on the DNA, as described in (29) (see Supplementary Figure S3). These results would suggest that the AP-lyase active site is located out of the central channel of *Bsu*Ku, as it occurs in human Ku70 (24). Therefore, the potential external placement of the AP-lyase active site, and the ‘noncanonical’ binding mode of *Bsu*Ku to a DNA without free ends could facilitate the recruitment of the protein to sites of DNA damage to exert its lyase activity.*B. subtilis* possesses three known AP endonuclease genes, *exoA*, expressed in growing cells and in the forespore compartment of the sporulating cell (49), *yqfs* (*nfo*) whose expression is under the control of the σ^F^ transcription factor and consequently is expressed late during sporulation (50) and *yshC* that codes for a family X DNA polymerase endowed with an AP endonuclease activity and whose expression pattern remains to be determined (51). Previous results indicated that *B. subtilis* spores lacking ExoA and/or Nfo showed an increased sensitivity to treatments that damage spore DNA through generation of AP sites and strand breaks. Those works advanced the potential importance of the BER pathway to repair the AP sites that accumulate during spore dormancy (49–50,52). However, besides an AP endonuclease, a dRP-lyase, a polymerase and a ligase should also be expressed during sporulation to reconstitute a complete BER pathway in the spore. The genes coding for *Bsu*Ku and *Bsu*LigD proteins form part of a regulon under the control of both, the RNA-polymerase sigma factor σ^G^ and the DNA-binding protein SpoVT whose expression is turned on in the forespore (53). These studies together with the finding of AP/5-dRP lyase that can act also on internal abasic sites (this work) could be indicative of a role of the NHEJ proteins in the BER pathway operating during spore outgrowth.

### Coordination of the AP/5′dRP- lyase activity of *Bsu*Ku and *Bsu*LigD polymerization and ligase activities during end joining

**Figure 6.**
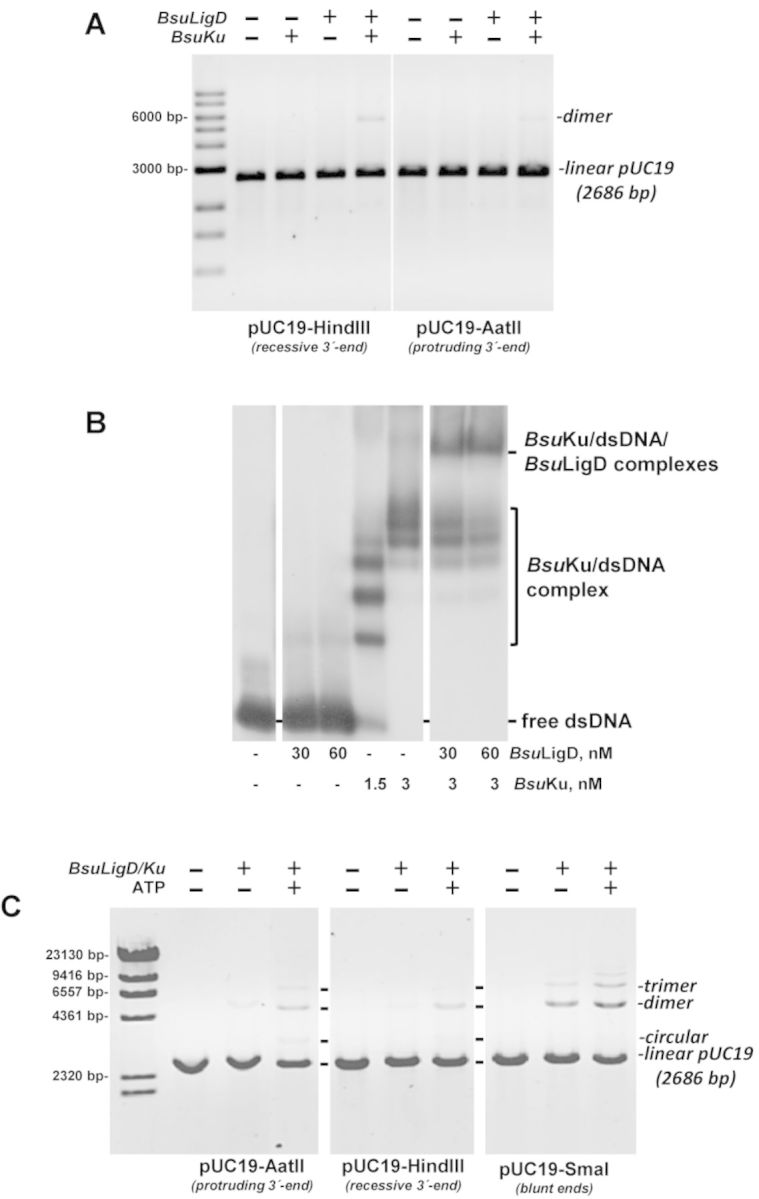
(**A**) End joining of linearized plasmids. The assay was performed as described in Materials and Methods by incubating 100 ng of the specified digested pUC19 plasmid with 0.6 mM MnCl_2_ in the absence (-) or presence (+) of 142 nM of *Bsu*LigD and *Bsu*Ku. After incubation for 60 min at 30°C, reactions were quenched by adjusting the mixtures to 0.2% SDS, 10 mM EDTA. Samples were incubated with 10 μg of proteinase K during 60 min at 37°C and further subjected to 0.7% agarose gel electrophoresis. The positions and size of DNA markers are indicated. (**B**) *Bsu*Ku recruits *Bsu*LigD to DNA ends. Reactions were performed as described in Materials and Methods by incubating 1 nM of a 216 bp DNA fragment and the indicated concentrations of *Bsu*LigD and *Bsu*Ku. For the supershift reactions, DNA was previously preincubated with *Bsu*Ku followed by the addition of the indicated amounts of *Bsu*LigD. (**C**) Effect of ATP on the end-joining of linearized plasmids. The assay was performed essentially as described in (A). When indicated, 142 nM of *Bsu*LigD/142 nM of *Bsu*Ku and/or 0.1 mM ATP were added.

Both eukaryotic and prokaryotic Ku have been described to bind dsDNA with high affinity (31,33,54). In the current working model of bacterial NHEJ, Ku recognizes and binds to the broken ends, recruiting LigD that further catalyzes both, filling of the gaps that could arise after synapsis and ligation of the resultant nick (4–5,55). To advance in the analysis of the AP-lyase activity of *Bsu*Ku during end-joining, we started analysing the ability of *Bsu*Ku/LigD complex to join nondamaged DNA ends, using as substrate a linearized pUC19 plasmid singly-digested with HindIII or AatII to obtain recessive or protruding 3′-ends, respectively. As shown in Figure 6A, *Bsu*Ku had not end-joining activity by itself, as expected, but its presence was absolutely necessary to allow *Bsu*LigD to join both, protruding and recessive 3′-ends, as deduced from the formation of dimers and as described in other bacterial members (27,33,56–57). These results suggest that *Bsu*Ku would be required to stabilize *Bsu*LigD binding to the termini of the DNA to guarantee the end-joining reaction. In this sense, previous studies showed the existence of a physical interaction between bacterial Ku and LigD at the ends of DNA molecules (31), similar to those described for eukaryotic Ku-Ligase IV/XRCC4 proteins (58). Therefore, to search for potential interactions between *Bsu*Ku and *Bsu*LigD, EMSAs assays with a radiolabeled dsDNA probe (216 bp) were performed. As shown in Figure 6B, whereas *Bsu*Ku led to the generation of several and stable Ku/DNA complexes, reflecting its high DNA binding efficiency and its ability to diffuse along DNA [as described for other Ku homologs (31,59–60)], *Bsu*LigD did not bind stably to the DNA substrate, accounting for the absence of ligation products described above. Conversely, addition of *Bsu*LigD to the preformed *Bsu*Ku–DNA complexes led to the generation of a stable DNA protein complex with a slower mobility than those formed by *Bsu*Ku alone. Thus, *Bsu*Ku would recruit *Bsu*LigD to the DNA termini endowing it with the binding stability required to rejoin and ligate two broken ends. Previous studies demonstrated that *Bsu*LigD is an ATP-dependent ligase (26). The ability of *Bsu*LigD to perform the ligation reaction in the absence of exogenous ATP (Figure 6A) is due to the proportion of the molecules that are already adenylated in the expression bacteria, as it has been reported to occur in other ATP-dependent DNA ligases (61–65). As shown in Supplementary Figure S4, the yield of ligated DNA in the ATP-independent ligation reaction increased linearly as a function of input *Bsu*LigD. The slope of the titration curve indicated that 0.37 fmol of the nicked DNA substrate was ligated per fmol of input BsuLigD. Considering that ATP-dependent ligation is a single-turnover reaction, we estimated that 37% of the *Bsu*LigD molecules contained AMP bound covalently at the ligation active site, a proportion similar to that obtained in other LigDs (64). Thereby, addition of 0.2 mM ATP enhanced the formation of dimers, trimers and, in the specific case of a blunt end plasmid (the SmaI-digested pUC19 plasmid) even tetramers (see Figure 6C). Under these experimental conditions, *Bsu*LigD–Ku complex also promoted intramolecular repair events, as deduced from the monomer circles among the reaction products.

**Figure 7.**
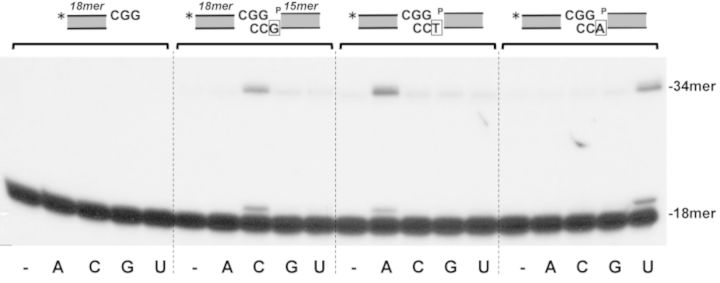
End joining of partially complementary 3′-protruding DNA ends. The assays were carried out as described in Materials and Methods by incubating 1.25 nM of the labeled hybrid sp1CGG/sp1c depicted on top of Figure in the presence of 71 nM of both *Bsu*LigD and *Bsu*Ku, 0.6 mM MnCl_2_, 2.5 nM of the nonlabeled hybrid (see Materials and Methods) indicated on top of Figure and 100 nM of the indicated NTP. The three DNA substrate molecules only differ in the marginal nucleotide indicated with open squares. After incubation for 30 min at 30°C, the elongation and ligation products were analysed by 8 M urea-20% PAGE and autoradiography. Asterisks indicate the 5′^32^P-labeled end of the primer strand.

Once established the competence of the *Bsu*LigD/*Bsu*Ku to carry out intermolecular ligation of complementary ends, we wondered whether such a complex could connect partially complementary 3′-protruding DNA ends coming from independent DNA molecules to form a ‘gap-like’ synaptic intermediate (66). Therefore, two hybrid molecules containing 3′ protruding DNA ends that differ in the nucleotide adjacent to the 5′-P were simultaneously incubated with the *Bsu*Ku–LigD complex (see scheme depicted in the top of Figure 7 note that only one of the molecules is 5′-labeled). The assay conditions were adjusted to prevent self-ligation of the labeled molecules (see Figure 7). As it is shown, 3′ extension of the labeled strand occurred specifically when the incoming ribonucleotide was complementary to the nucleotide adjacent to the 5′-P of the nonlabeled molecule (Figure 7). This reaction was absolutely dependent on the presence of *Bsu*Ku (not shown), indicating that synapsis between the two molecules took place. After addition of the complementary nucleotide, the resulting nick was sealed by the ligase activity to render a repaired (ligated) product. Thus, these results show the ability of *Bsu*Ku–LigD complex to mediate the bridging of two partially complementary DNA ends, generating a minimum gap-like synaptic complex that is filled and subsequently sealed by the polymerization and ligase activities, mimicking a DSB repair reaction.

**Figure 8.**
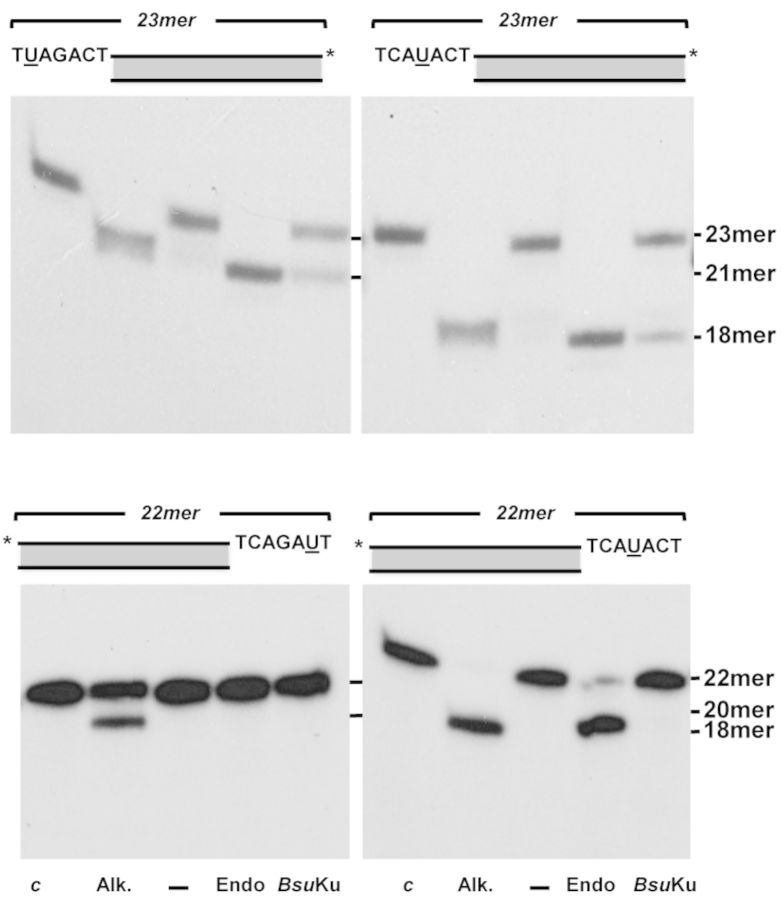
*Bsu*Ku AP-lyase on protruding ends. The assay was performed as described in Materials and Methods. The substrates containing a uracil at the specified position within the 5′- (upper panels) or 3′- (lower panels) protruding ends were incubated with *Escherichia coli* UDG (*c*) to create an AP site in nearly all DNA molecules. After incubation of the AP-containing molecules with 2 units of *E. coli* Endo III (Endo), or 142 nM *Bsu*Ku for 30 min at 30°C, samples were analysed by 8 M urea-20% PAGE and autoradiography, as described in Materials and Methods. Positions corresponding to the products are indicated. Asterisks indicate either the ^32^P-5′ or the [^32^P]3′dAMP-3′end.

As shown in Figure 8, *Bsu*Ku AP-lyase was also active on AP sites placed within a protruding 5′ end, whereas its activity was negligible on 3′ overhangs like human Ku (25). This fact could be relevant in an end-joining context, as nicking at a protruding 3′ end would generate a 3′-PUA terminus that should be sanitized by additional nucleolytic activities before ligation. Contrarily, nicking within a 5′ protruding end would release an ssDNA portion with a ligatable 5′P terminus.

**Figure 9.**
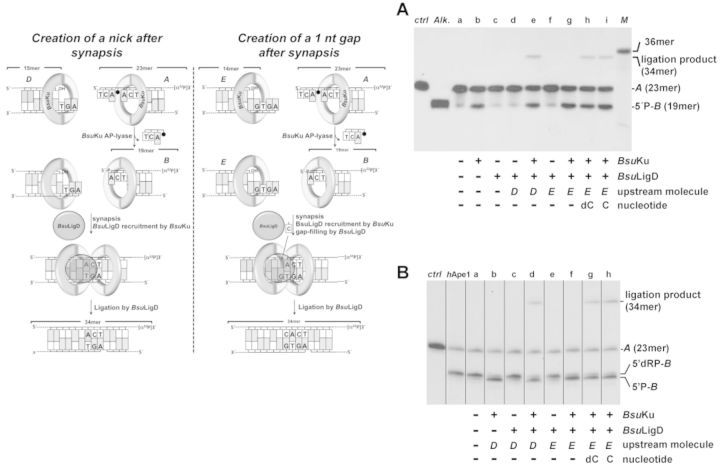
End joining of partially complementary DNA ends with near terminal AP sites. *Left panel*, schematic representation of the end-joining reaction, indicating the different substrates (filled circle represents an abasic site), products and the catalytic reactions that take place (see main text). (**A**) End joining of partially complementary DNA ends with near terminal AP sites; the assay was carried out as described in Materials and Methods by incubating 0.7 nM of the 3′-labeled substrate A and, when indicated 114 nM of *Bsu*Ku, 114 nM *Bsu*LigD, 10 μM of the indicated nucleotide and 1 nM of the specified upstream substrate. After incubation for 30 min at 30°C, the products of the reaction were analysed by 8 M urea-20% PAGE and autoradiography (*M*: 36mer oligonucleotide used as size marker; *ctrl*: DNA after incubation with *E. coli* UDG; *Alk*. alkaline hydrolysis of the UDG-treated DNA). (**B**) End joining with a downstream DNA molecule bearing a protruding 5′-dRP end; the assay was performed as described in Materials and Methods by incubating 0.7 nM of the 3′-labeled substrate A with *h*APE1. When indicated, the digested substrate was incubated with 114 nM of *Bsu*Ku, 114 nM of *Bsu*LigD, 10 μM of the indicated nucleotide and 1 nM of the specified upstream substrate for 30 min at 30°C. After incubation, the products of the reaction were analysed by 8 M urea-20% PAGE and autoradiography. The lengths of the labeled substrate and the degradation and ligation products are indicated. Figure 9B is a composite image made from different parts of the same experiment.

Once proved the capacity to perform end-joining of nondamaged DNA ends, we assessed the ability of the *Bsu*Ku–LigD complex to join substrates that contain AP sites proximal to their 5′ protruding ends (see scheme in Figure 9). The labeled downstream molecule *A* harbors a AP site containing 5′-protruding end that is solely partially complementary to unlabeled upstream substrates *D* (left panel of scheme) and *E* (right panel of scheme). Only after precise incision at the AP site in molecule *A*, the resultant molecule *B* would harbor a 5′ protruding end so that synapsis to substrates *D* and *E* would render a nicked and a 1nt gapped molecule, respectively. Figure 9A shows that, as expected, *Bsu*Ku incises at the AP site of molecule *A* (23mer) to produce the 19mer molecule *B* (lane *b*) that is not used as self-ligatable substrate by *Bsu*LigD (lane *c*). In contrast, simultaneous addition of substrates *A* and either *D* or *E* gave rise to a 34mer ligation product that results from the incision of *A* by *Bsu*Ku, synapsis to either *D* or *E* and final sealing by *Bsu*LigD. Importantly, when molecule *E* was used as the upstream substrate, the 1 nt gapped molecule that resulted after synapsis had to be filled by *Bsu*LigD before the ligation step (lanes *g*, *h* and *i*).

Several genotoxicants, as reactive oxygen species and ionizing radiation generate a variety of DNA damage including AP sites. *In vitro* and *in vivo* studies in *E. coli* (67,68), as well as in mammalian cells (69) have shown that attempted repair of two closely opposed AP sites can result in the formation of DSBs due to the introduction of single-stranded breaks by the cellular AP endonucleases. In mammalian cells, the DNA can be degraded or inaccurately repaired by Ku-independent pathways as single-strand annealing and microhomology-mediated end joining, or by the Ku-dependent NHEJ (69). Therefore, we analysed *in vitro* the ability of the *Bsu*Ku–LigD complex to perform end joining on these kind of molecules. As it can be observed in Figure 9B, when the AP site of molecule *A* is hydrolyzed by an AP endonuclease (lane *a*), the resulting 5′dRP moiety is removed by *Bsu*Ku (lanes *b*, *d* and *f*) to allow the final sealing step by *Bsu*LigD (lanes *d*, *g* and *h*), rendering a repaired product. These results suggest that the *Bsu*Ku–LigD complex has the potentiality to repair efficiently the DSBs produced by endonucleolytic hydrolysis of clustered AP sites during spore outgrowth.

## CONCLUSION

Although initially described in human Ku, the presence of an AP-lyase in bacterial homologs could not be anticipated as these members lack the N-terminal von Willebrand A domain of the eukaryotic Ku70 where the lysine responsible for this activity resides. To our knowledge, this is the first report of the presence of an AP-lyase activity in a bacterial Ku factor. Its ability to act on ssDNA, nicked molecules and on circular DNAs, as well as its functional coordination with an AP endonuclease and its natural partner, *Bsu*LigD, makes this protein able to cooperate in processing of AP sites during the NHEJ pathway. Our results show that this activity is not restricted to *B. subtilis* as is also present in the Ku protein of the phylogenetically distant bacterium *P. aeruginosa*, allowing us to expand our observations to other bacterial members provided with an NHEJ system.

## SUPPLEMENTARY DATA

Supplementary data are available at NAR Online.

SUPPLEMENTARY DATA
