# Supplementary material for: Efficient processing of abasic sites by bacterial nonhomologous end-joining Ku proteins
Source: Nucleic Acids Res. 2014 Oct 29;42(21):13082–95. doi: 10.1093/nar/gku1029 (PMC4245934; doi:10.1093/nar/gku1029)
Supplement: SUPPLEMENTARY DATA [file supp_42_21_13082__index.html]

Efficient processing of abasic sites by bacterial nonhomologous end-joining Ku proteins — SUPPLEMENTARY DATA 

# Efficient processing of abasic sites by bacterial nonhomologous end-joining Ku proteins

## SUPPLEMENTARY DATA

**Files in this Data Supplement:**

- SUPPLEMENTARY DATA
