# Supplementary material for: Efficient processing of abasic sites by bacterial nonhomologous end-joining Ku proteins
Source: Nucleic Acids Res. 2014 Oct 29;42(21):13082–95. doi: 10.1093/nar/gku1029 (PMC4245934; doi:10.1093/nar/gku1029)
Supplement: SUPPLEMENTARY DATA [file supp_gku1029_nar-00866-d-2014-File011.pdf]

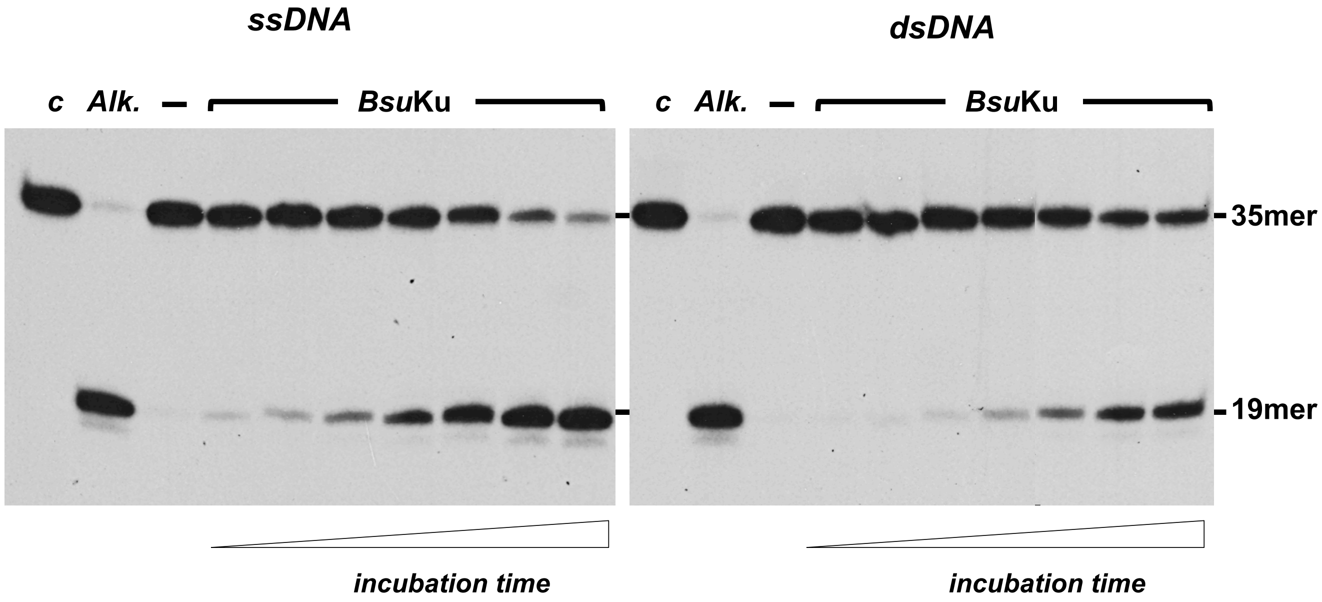

Supplementary Figure S1. *Bsu*Ku AP-lyase activity on ss- and dsDNA containing an abasic site. Either the 3´[^{32}P]3´dAMP labeled 35mer oligonucleotide pU containing a uracil at position 16 (ssDNA) or hybridized to the complementary oligonucleotide pG (dsDNA) was treated with *E. coli* UDG to leave an intact AP site in nearly all DNA molecules (*Alk*. alkaline hydrolysis of the UDG-treated DNA). After incubation of the AP-containing molecules with *Bsu*Ku (228 nM) for 4, 8, 15, 30, 60, 120 and 180 min at 30 ºC, samples were analyzed by 8 M urea-20% PAGE and autoradiography, as described in Materials and Methods. Position corresponding to product (19mer) is indicated.

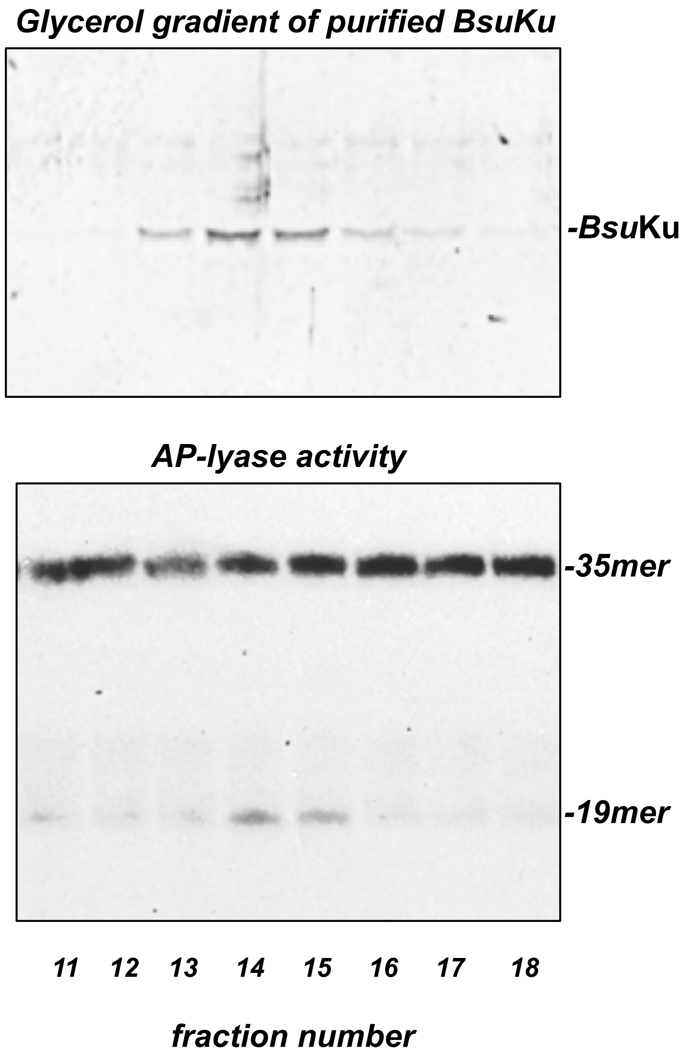

Supplementary Figure S2. AP-lyase activity is intrinsic to *Bsu*Ku. The [^{32}P]3´dAMP-3´end labeled 35mer oligonucleotide containing an uracil at position 16 was treated with *E. coli* UDG, leaving an intact AP site. The resulting AP-containing DNA was incubated for 2 h at 30°C with 5 µl of fractions 11–18 obtained after sedimentation of the purified *Bsu*Ku on a 15–30% glycerol gradient. After incubation reactions were stopped by adding 100 mM $NaBH_4$ and further analyzed by 8 M urea-20% PAGE and autoradiography. Position of products is indicated.

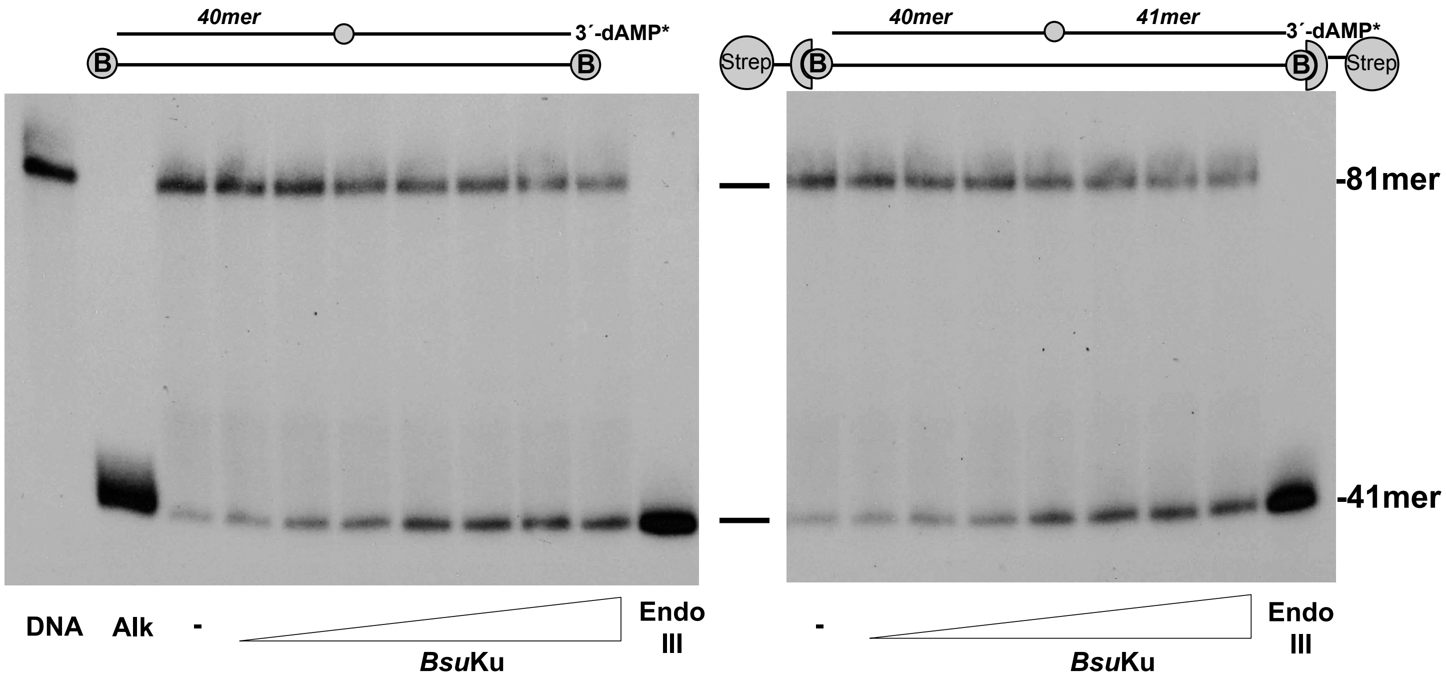

Supplementary Figure S3. *Bsu*Ku AP-lyase activity on a single internal abasic site. The 3´[^{32}P]3´dAMP labeled 81mer oligonucleotide SU containing a uracil at position 40 was hybridized to the complementary 3´- and 5´ biotinilated oligonucleotide SU-compl (see Materials and Methods). 3.2 nM of the hybrid molecule was preincubated for 5 min at 4 ºC in the absence (left panel) or presence (right panel) of 200 nM Strptavidine. The substrate was further treated with *E. coli* UDG to leave an intact AP site in nearly all DNA molecules (*Alk*. alkaline hydrolysis of the UDG-treated DNA). The resulting AP-containing DNA was incubated with increasing amounts of *Bsu*Ku (0, 3.2, 6.2, 12.5, 25, 50, 100 and 200 ng) at 30 ºC for 1 h. Reactions were stopped by addition of 10 µg of proteinase K and further incubation at 50 ºC for 1 h in the presence of 0.4% SDS. Reactions were analyzed by 8 M urea–20% PAGE and autoradiography. Position of products is indicated.

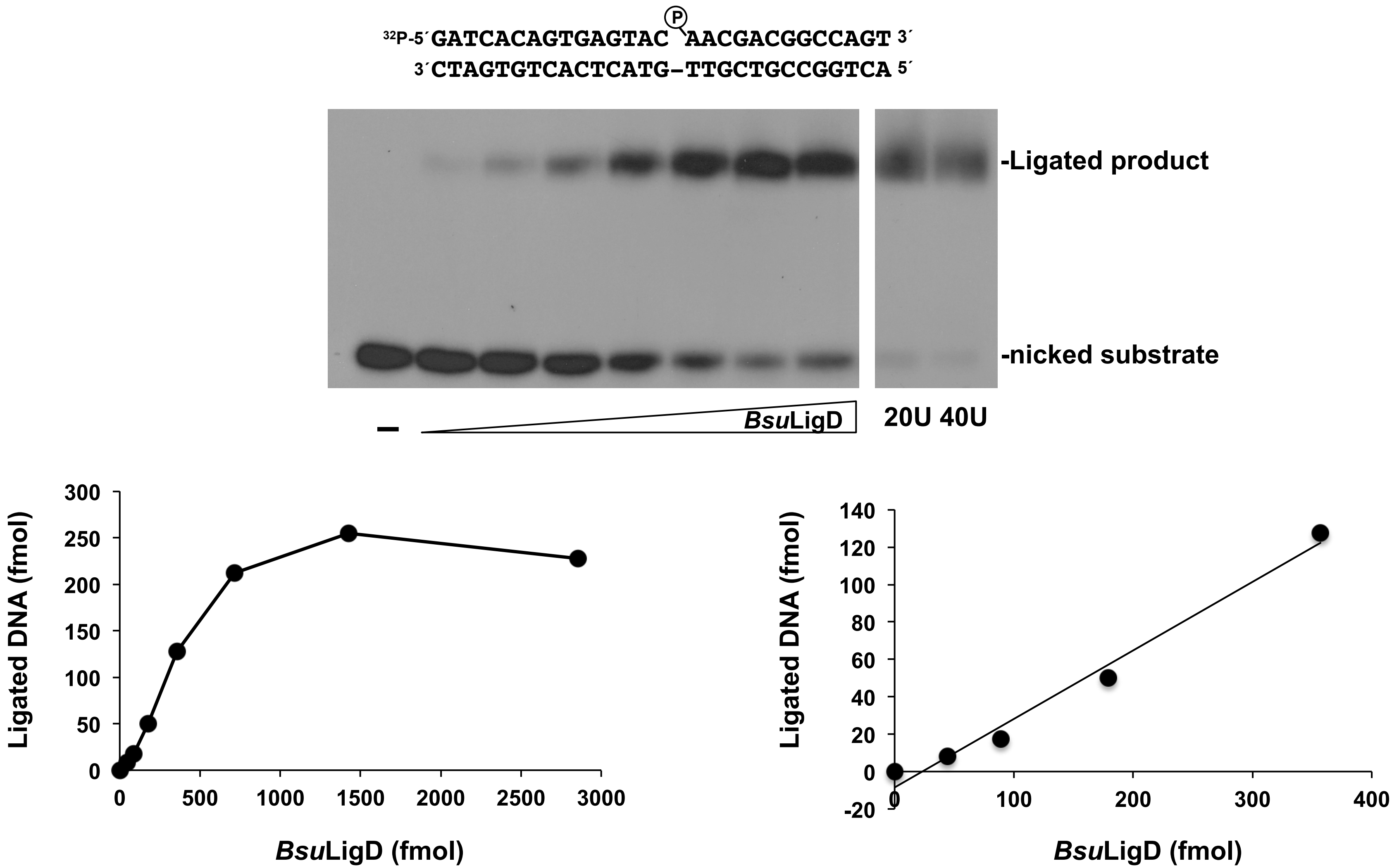

Supplementary Figure S4. ATP-independent sealing reaction. The incubation mixture contained, in a final volume of 12.5 µl, 12 mM Tris-HCl, pH 7.5, 1 mM EDTA, 20 mM ammonium sulphate, 0.1 mg/ml BSA, 40 µM $MnCl_2$, 300 fmols of the nicked substrate and increasing amounts of *Bsu*LigD (45, 89, 179, 357, 714, 1428, 2856 fmols). After incubation for 6 h at 30 ºC, the reactions were stopped by adding EDTA up to 10 mM. Samples were analyzed by 8 M urea, 20% PAGE and autoradiography. Estimation of the proportion of adenylated BsuLigD was performed by plotting the ligation product yielded against *Bsu*LigD amounts in the linear range and further linear regression with the Kaleidagraph 3.6.4 software. Determination of the proportion of non-ligatable substrate was performed by incubating the nicked substrate with 20 and 40 units of T4 DNA ligase (NEB) in its corresponding reaction buffer for 3 h at 37 ºC. The propotion of adenylated *Bsu*LigD molecules was estimated from the slope (bottom, right) of the titration curve (bottom, left).
